# Confirmation of the distribution of *Gonyosoma prasinum* (Blyth, 1855) and *Boiga multomaculata septentrionalis* Köhler, Charunrochana, Mogk, Than, Kurniawan, Kadafi, Das, Tillack & O’Shea, 2023 (Serpentes, Colubridae) in China

**DOI:** 10.3897/BDJ.14.e177584

**Published:** 2026-03-26

**Authors:** Shuo Liu, Mian Hou, Mamai Yue, Anru Zuo, Maolin Yan, Changsheng Zuo, Zhengpan Duan, Fawang Yin, Song Li

**Affiliations:** 1 Yunnan Key Laboratory of Biodiversity Information, Kunming Institute of Zoology, Chinese Academy of Sciences, Kunming, China Yunnan Key Laboratory of Biodiversity Information, Kunming Institute of Zoology, Chinese Academy of Sciences Kunming China; 2 Kunming Natural History Museum of Zoology, Kunming Institute of Zoology, Chinese Academy of Sciences, Kunming, China Kunming Natural History Museum of Zoology, Kunming Institute of Zoology, Chinese Academy of Sciences Kunming China; 3 Sichuan Normal University, Chengdu, China Sichuan Normal University Chengdu China; 4 Yunnan Tongbiguan Provincial Nature Reserve Management and Protection Bureau, Yingjiang, China Yunnan Tongbiguan Provincial Nature Reserve Management and Protection Bureau Yingjiang China

**Keywords:** cytb, morphology, phylogeny, snake, Yunnan

## Abstract

**Background:**

*Gonyosoma prasinum* (Blyth, 1855) was once considered a widely distributed species. Later, it was revealed that it is a species complex and has been divided into three species. Presently, *Gonyosoma prasinum* (Blyth, 1855) is known to be only distributed in India, Myanmar and Bhutan and there is no confirmed record of its occurrence in China.

*Boiga multomaculata septentrionalis* Köhler, Charunrochana, Mogk, Than, Kurniawan, Kadafi, Das, Tillack & O’Shea, 2023 is a subspecies of *B. multomaculata* described from Kachin State, Myanmar. It is known to be distributed in Myanmar and India and is speculated to be also distributed in China.

**New information:**

Recently, two snake specimens were collected from Yingjiang County, Dehong Prefecture, Yunnan Province, China. Based on morphological comparison and phylogenetic analysis, we determine that the two specimens belong to *Gonyosoma prasinum* and *Boiga multomaculata septentrionalis*, respectively. Therefore, we confirm the distribution of these two species in China.

## Introduction

Previously, *Gonyosoma prasinum* (Blyth, 1855) was considered to be widely distributed in India, Bhutan, Myanmar, Thailand, Malaysia, Laos, Vietnam and China, until Liu et al. (2021) described *G. coeruleum* Liu, Hou, Lwin, Wang & Rao, 2021 from Yunnan Province, China, which was previously confused with *G. prasinum*. Subsequently, David et al. (2022) refined the distribution ranges of *G. prasinum* and *G. coeruleum*. Wallach et al. (2024) divided the genus *Gonyosoma* into five genera, namely *Gonyophis* Boulenger, 1891, *Gonyosoma*, *Rhadinophis* Vogt, 1922, *Rhynchophis* Mocquard, 1897 and *Verdigrophis* Wallach, Midtgaard & Hsiao, 2024 and they proposed the tribe *Gonyosomini* Wallach, Midtgaard & Hsiao, 2024 to include the five genera. However, the division of the genus *Gonyosoma* has not been widely recognised. Poyarkov et al. (2025) retained *Gonyosoma* as a monotypic genus and regarded *Gonyophis*, *Rhadinophis*, *Rhynchophis* and *Verdigrophis* as the junior synonyms of *Gonyosoma*. In addition, Poyarkov et al. (2025) described another species in the *G. prasinum* complex, *G. iadinum* Poyarkov, Bragin, Idiiatullina, Tran, Le, David & Nguyen, 2025, from Vietnam and Laos. So far, *G. prasinum* was confirmed to be distributed in India, Bhutan and Myanmar, *G. coeruleum* was known to be distributed in China, Laos, Vietnam, Myanmar, Thailand and Malaysia, and *G. iadinum* was known to be distributed in Laos and Vietnam (Liu et al. 2021, David et al. 2022, Poyarkov et al. 2025). However, it is uncertain whether *G. prasinum* is distributed in China. Liu et al. (2021) speculated that *G. prasinum* may be distributed in south-eastern Xizang Autonomous Region, China, which borders India and Myanmar.

*Boiga multomaculata* (Boie, 1827) and *B. ochracea* (Theobald, 1868) were once considered as two different species (Wallach et al. 2014). Köhler et al. (2023) conducted a taxonomic revision of *B. multomaculata* and *B. ochracea*, they regarded *B. ochracea* as a subspecies of *B. multomaculata* and described a new subspecies of *B. multomaculata*. Thus, currently, *B. multomaculata* contains three subspecies, namely *B. m. multomaculata*, *B. m. ochracea* and *B. m. septentrionalis* Köhler, Charunrochana, Mogk, Than, Kurniawan, Kadafi, Das, Tillack & O’Shea, 2023. According to Köhler et al. (2023), *B. m. multomaculata* is distributed in Indonesia, Thailand, Laos, Cambodia, Vietnam and China, *B. m. ochracea* is distributed in India, Myanmar, Bangladesh, Thailand and Laos and *B. m. septentrionalis* is distributed in Myanmar and India. Köhler et al. (2023) considered that *B. m. septentrionalis* is likely to occur in China, but further confirmation is needed.

During our field surveys in western Yunnan Province, China, in 2025, two snake specimens were collected from Yunnan Tongbiguan Provincial Nature Reserve in Yingjiang County, Dehong Prefecture. Detailed morphological comparisons and molecular analysis indicated that these two specimens belong to *Gonyosoma prasinum* and *Boiga multomaculata septentrionalis*, respectively. Therefore, we confirm the distribution of *G. prasinum* and *B. m. septentrionalis* in China and provide descriptions of the two specimens from China.

## Materials and methods

### Sampling

**Figure 1.**
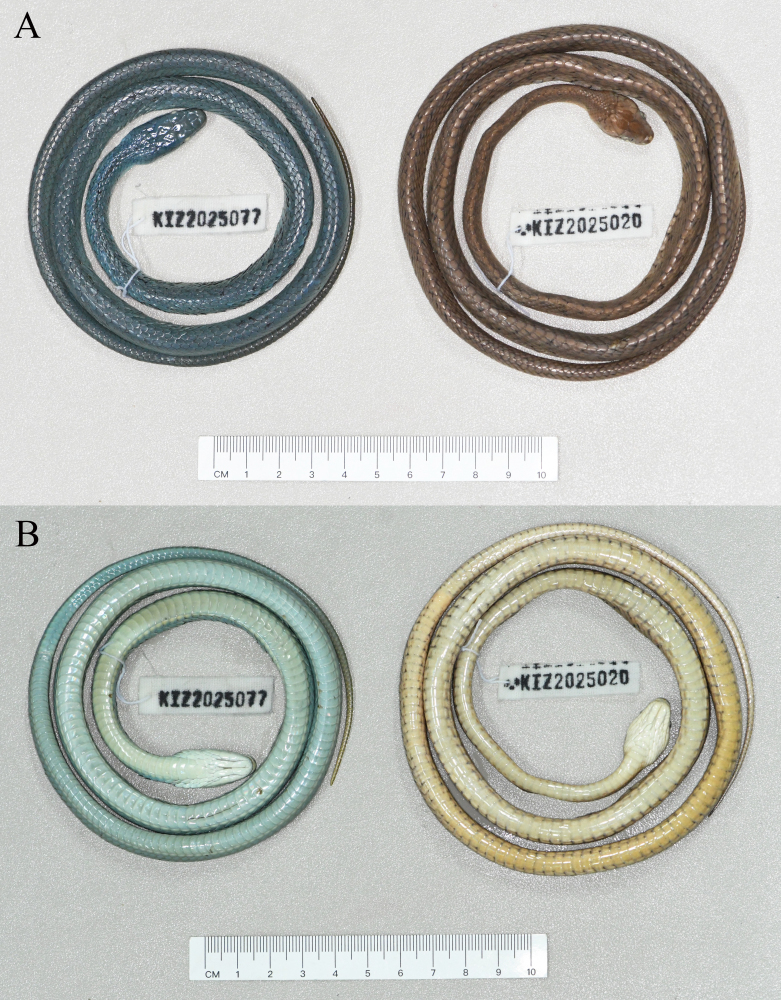
The specimens of *Gonyosoma prasinum* (KIZ2025077) and *Boiga multomaculata septentrionalis* (KIZ2025020) from China in preservative. **A** dorsal view; **B** ventral view.

Field surveys were conducted under the permit of Yunnan Tongbiguan Provincial Nature Reserve Management and Protection Bureau. Two specimens were collected from Kachang and Xima towns, Yingjiang County, Dehong Prefecture, Yunnan Province, China, respectively (Fig. 1). After being photographed and euthanised and then preserved in approximately 75% ethanol, they were deposited at Kunming Natural History Museum of Zoology, Kunming Institute of Zoology, Chinese Academy of Sciences (KIZ).

### Molecular analysis

Genomic DNA was extracted from liver tissues taken from the specimens. A fragment of the mitochondrial cytochrome b gene (cytb) was amplified and sequenced. Primers and molecular experimental conditions were referenced from Poyarkov et al. (2025). New sequences were deposited in GenBank. Homologous and outgroup sequences were obtained from GenBank (Tables 1, 2).

Sequences were aligned using ClustalW (Thompson et al. 1994) with default parameters. Bayesian Inference (BI) and Maximum Likelihood analysis (ML) were used to construct the phylogenetic tree. The technical computation methods for genetic distance calculating and BI and ML analyses were the same as those used in Liu et al. (2025).

### Morphological characteristics

Morphological terminology followed Poyarkov et al. (2025): snout-vent length (SVL), from the snout to the last ventral scale; tail length (TaL), from the anal to the tail tip; total length (TL), from the tip of the snout to the end of the tail; head length (HL), from the posterior edge of the jaw to the tip of the snout; head width (HW), maximum head width; eye diameter (ED), maximum vertical eye diameter; loreals (Lor); preoculars (PrO); postoculars (PoO); supralabials (SL); infralabials (IL); supralabials in contact with the eye (SL-E); anterior temporals (AT); posterior temporals (PT); dorsal scale rows at the neck (ASR); dorsal scale rows at the mid-body (MSR); dorsal scale rows before the vent (DSR); keeled dorsal scale rows at the neck (KAD); keeled dorsal scales at the mid-body (KMD); keeled dorsal scale rows before the vent (KPD); condition of the cloacal plate (CLP); ventrals (VEN); and subcaudals excluding the terminal scute (SC).

## Taxon treatments

### Gonyosoma prasinum

(Blyth, 1854)

168559E2-6D28-5FA0-9425-BBB840A2430A

#### Materials

- **Type status:** Other material. **Occurrence:** catalogNumber: KIZ2025077; individualCount: 1; sex: male; lifeStage: adult; occurrenceID: 20E878FF-499C-5EE8-BAEF-3A745DF8D05A; **Location:** country: China; stateProvince: Yunnan; locality: Kachang Township, Yingjiang county, Dehong Prefecture; verbatimElevation: 720 m; verbatimCoordinates: 25°1′28″ N 97°43′30″ E; **Event:** eventRemarks: collected on 31 July 2025 by Fawang Yin; **Record Level:** basisOfRecord: preserved specime

#### Description of the specimen from China

Adult male, body slender, SVL 558 mm, TaL 159 mm, TaL/TL 0.22; head elongate, HL 24.0 mm, distinct from neck, flattened, distinctly longer than wide, HL/HW 2.02, narrowed anteriorly; snout long, slightly projecting beyond lower jaw; nostril relatively small, lateral, nasal divided into two scales, anterior one slightly larger than posterior one; eye large, ED 3.9 mm, ED/HL 0.16, pupils round; rostral approximately triangular, wider than high, visible from above; two internasals, nearly square in shape, bordered by two prefrontals posteriorly; supraocular one on each side, large; frontal pentagonal, longer than wide, narrowed posteriorly; parietals large, longer than wide, in contact with each other; loreal one on each side, small, wider than high; preocular one on each side, large; postoculars two on each side, lower ones smaller; anterior temporal two on left side and one on right side, posterior temporal two on left side and one on right side; supralabials nine on each side, first and second in contact with nasal, second and third in contact with loreal, fourth to sixth entering orbit, eighth largest; infralabials ten on each side, first pair contact with each other, first to fifth in contact with anterior pair of chin shields; two pairs of large, elongate chin shields, posterior pair longer than anterior pair; Dorsal scales in 19-19-13 rows, middle 3–5 rows feebly keeled; ventrals 207 with a lateral keel; subcaudals 99, paired; cloacal plate undivided.

#### Colouration in life

**Figure 2.**
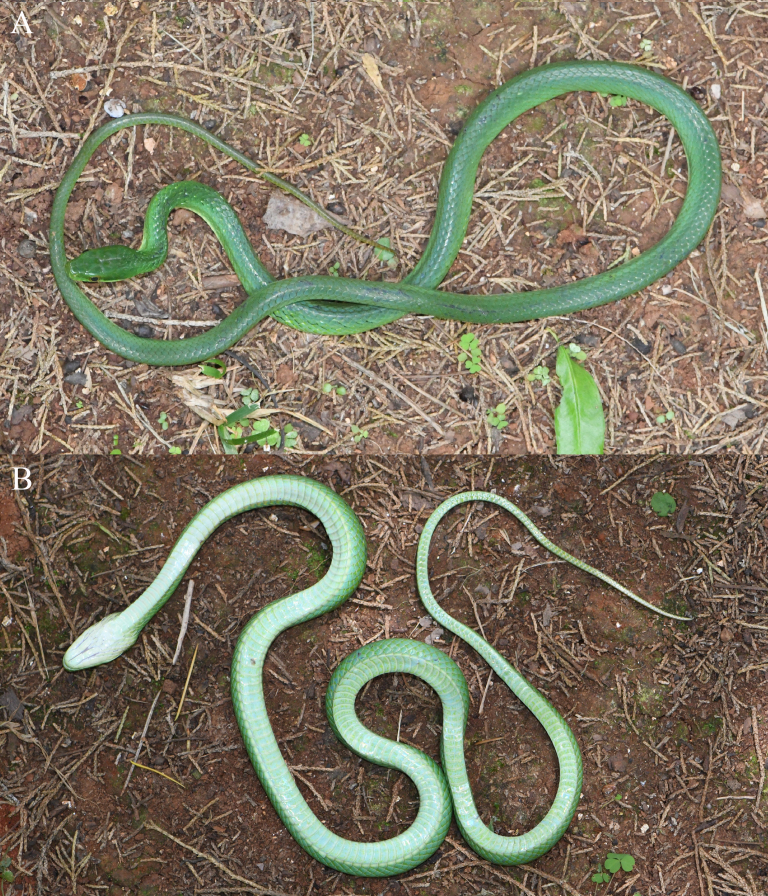
*Gonyosoma prasinum* (specimen KIZ2025077) from China in life. **A** dorsal view; **B** ventral view.

**Figure 3.**
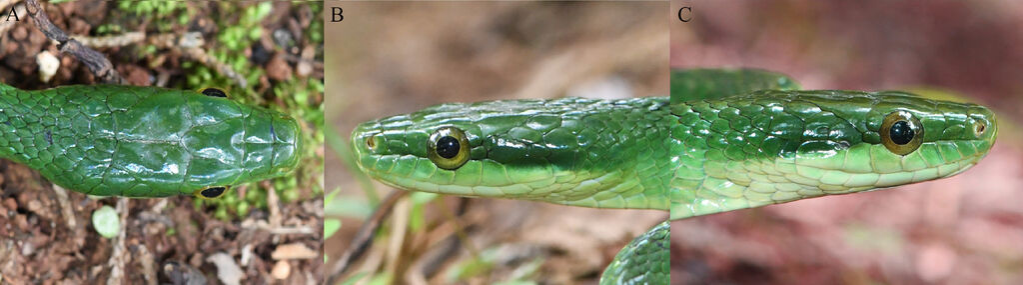
Close-up views of the head of *Gonyosoma prasinum* (specimen KIZ2025077) from China in life. **A** Dorsal view; **B** left view; **C** right view.

Dorsal surface green with yellow-brownish tail tip, reticulate pattern consisting of yellow, black and white on interstitial skin; upper lips light green to greenish-white; anterior ventral surface white to greenish-white, posterior ventral surface greenish-white to light-green; ventral surface of tail tip brownish-yellow; a yellowish-white ventrolateral strip on each side from neck to before vent; iris greenish-yellow (Figs 2, 3).

### Boiga multomaculata septentrionalis

Köhler, Thammachoti, Mogk, Than, Tillack, Kurniawan, Kadafi, Das & O'Shea, 2023

54C46B13-E0AC-51B3-AEF1-2FB900A2B08F

#### Materials

- **Type status:** Other material. **Occurrence:** catalogNumber: KIZ2025020; individualCount: 1; sex: male; lifeStage: adult; occurrenceID: B4E877DA-3FAA-5854-966D-6740184D556D; **Location:** country: China; stateProvince: Yunnan; locality: Xima Township, Yingjiang county, Dehong Prefecture; verbatimElevation: 1670 m; verbatimCoordinates: 24°46′48″ N 97°40′41″ E; **Event:** eventRemarks: collected on 28 March 2025 by Fawang Yin; **Record Level:** basisOfRecord: preserved specime

#### Description of the specimen from China

Adult male, body slender, SVL 870 mm, TaL 166 mm, TaL/TL 0.16; head large, significantly distinct from neck, longer than wide, HL/HW 1.58, distinctly narrowed anteriorly; snout relatively short, projecting beyond lower jaw; nostril large, lateral, nasal divided into two scales, subequal in size; eye large, ED 3.7 mm, ED/HL 0.18, pupils vertical; rostral approximately triangular, slightly wider than high, slightly visible from above; two internasals, nearly triangular in shape, bordered by two prefrontals posteriorly; supraocular one on each side, triangular in dorsal view; frontal pentagonal, wide almost equal to length; parietals large, longer than wide, in contact with each other; loreal one on each side, small; preocular one on each side, vertically elongated; postoculars two on each side; anterior temporal two on each side, posterior temporal two on each side; supralabials eight on each side, first and second in contact with nasal, second and third in contact with loreal, third to fifth entering orbit, seventh largest; infralabials 11 on each side, first pair contact with each other, first to fifth in contact with anterior pair of chin shields; two pairs of large chin shields, two pairs approximately equal in length; Dorsal scales in 19-19-15 rows, all smooth; ventrals 222; subcaudals 96, paired; cloacal plate undivided.

#### Colouration in life

**Figure 4.**
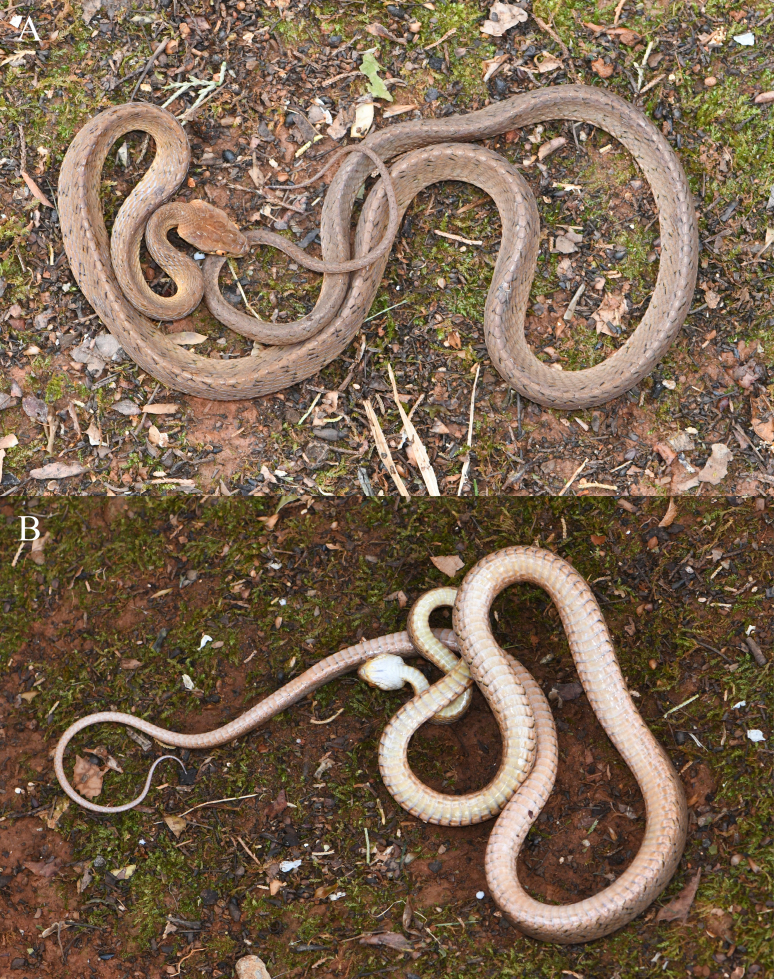
*Boiga multomaculata septentrionalis* (specimen KIZ2025020) from China in life. **A** dorsal view; **B** ventral view.

**Figure 5.**
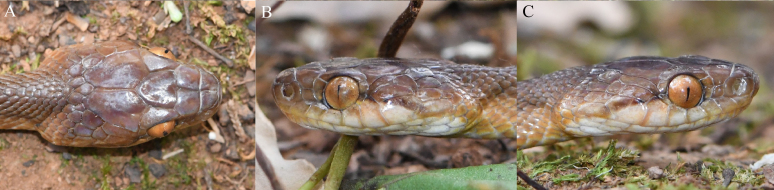
Close-up views of the head of *Boiga multomaculata septentrionalis* (specimen KIZ2025020) from China in life. **A** Dorsal view; **B** left view; **C** right view.

Dorsal surface of head light brown, dorsal surface of body and tail light greyish-brown with many narrow and short black strips; upper lips light brown to yellowish-white; ventral surface of head white, anterior ventral surface of body yellowish-white, posterior ventral surface of body and tail pinkish-white; iris brass-coloured (Figs 4, 5).

## Analysis

**Figure 6.**
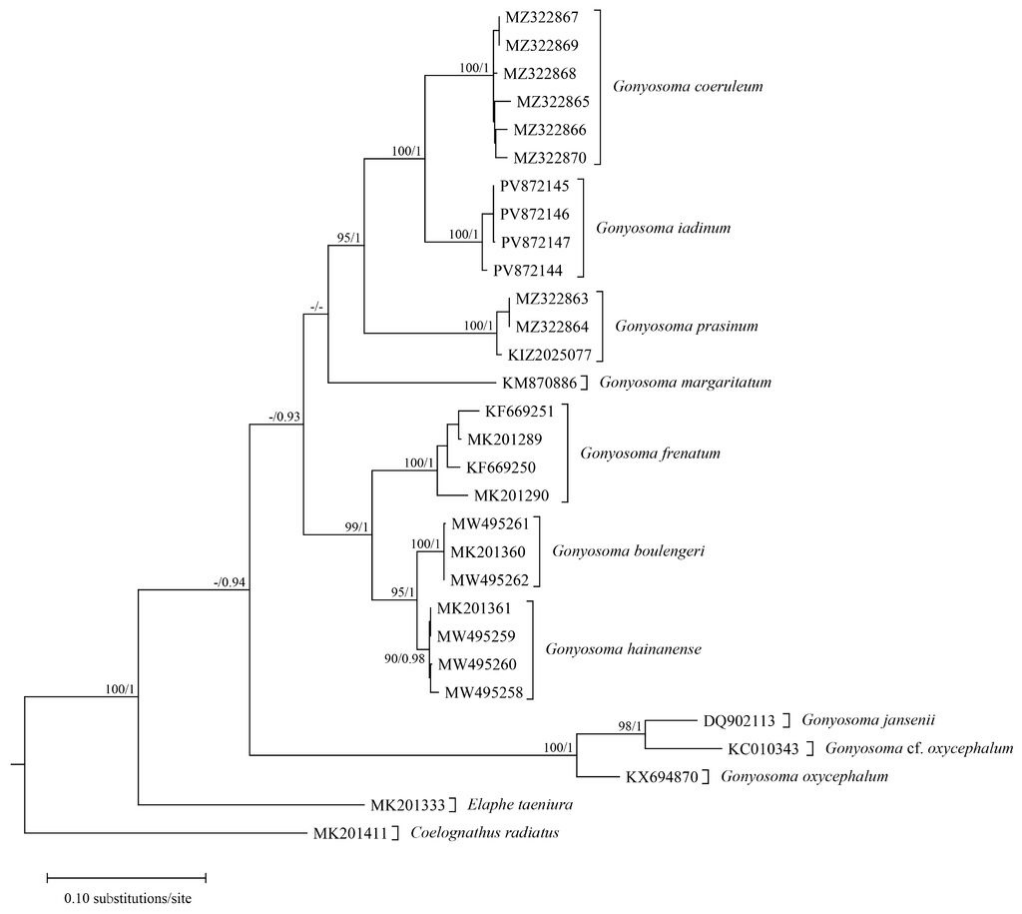
Maximum Likelihood phylogenetic tree of the genus *Gonyosoma*, based on cytb sequences. Numbers after and behind the “/” are Maximum Likelihood ultrafast bootstrap values and Bayesian posterior probabilities, respectively. “-” represents values less than 90/0.90.

**Figure 7.**
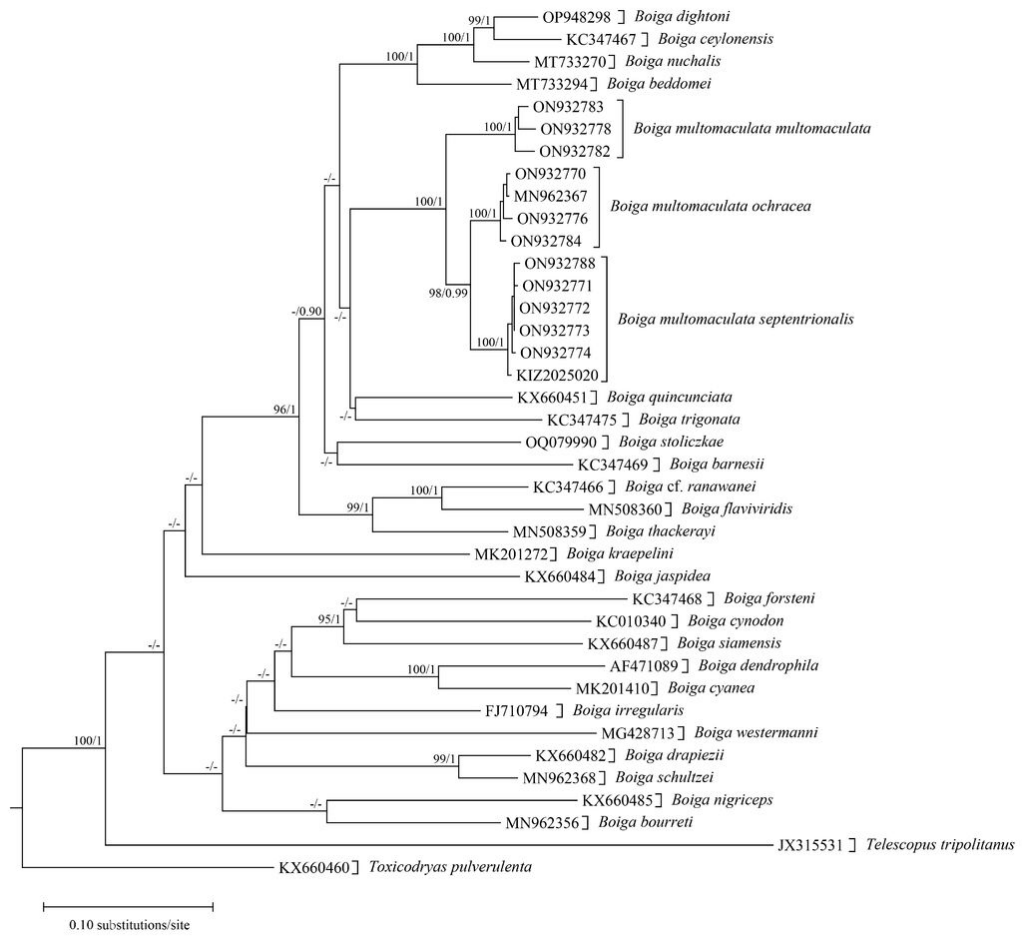
Maximum Likelihood phylogenetic tree of the genus *Boiga* based on cytb sequences. Numbers after and behind the “/” are Maximum Likelihood ultrafast bootstrap values and Bayesian posterior probabilities, respectively. “-” represents values less than 90/0.90.

Genetically, the two specimens from Kachang and Xima towns clustered with *Gonyosoma prasinum* and *Boiga multomaculata septentrionalis* both with strong support, respectively (Figs 6, 7). The genetic distance (uncorrected p-distance) between the specimen from Kachang Town and other specimens of *G. prasinum* was only 1.2% and the genetic distance (uncorrected p-distance) between the specimen from Xima Town and other specimens of *B. m. septentrionalis* was only 0.6%.

Morphologically, the specimen from Kachang Town agrees with the diagnosis of *Gonyosoma prasinum* in having greenish-yellow iris and an entire cloacal plate and the specimen from Xima Town agrees with the diagnosis of *Boiga multomaculata septentrionalis* in having a light-brown dorsal colour with almost no pattern, 222 ventrals and 96 subcaudals (Table 3).

Combining the results of molecular and morphological analysis, we determined that the two specimens from Yingjiang County, Dehong Prefecture, Yunnan Province, China, belong to *Gonyosoma prasinum* and *Boiga multomaculata septentrionalis*, respectively.

## Discussion

It is doubtful whether *Gonyosoma prasinum* is distributed in China since *G. prasinum* sensu lato was split into multiple species. There are low-altitude forests in the south-eastern Xizang Autonomous Region, China, which borders India and Myanmar (Che et al. 2020) and the discovery of *G. prasinum* in Xizang is expected. However, there is no record of *G. prasinum* in Xizang so far (Guo and Che 2024). Through this study, we confirm the distribution of *G. prasinum* in Yunnan Province, China. As for whether *G. prasinum* is distributed in Xizang, more field surveys are needed to determine its existence.

Dehong Prefecture is the westernmost part of Yunnan Province, China. Previously, it was known from literature that *Gonyosoma coeruleum* is distributed in Dehong Prefecture (Yang and Rao 2008, David et al. 2022, Poyarkov et al. 2025). This study demonstrates that both *G. coeruleum* and *G. prasinum* are distributed in Dehong Prefecture. However, no sympatric distribution of these two species has been found. *Gonyosoma coeruleum* is distributed almost throughout the prefecture, but is usually found in areas above 1,000 m in altitude, while *G. prasinum* seems to be distributed only in a few parts of the prefecture close to the national border and generally inhabits areas below 1,000 m in altitude. In addition, according to David et al. (2022) and Poyarkov et al. (2025), there are differences in hemipenial morphology of these two species. Therefore, it is speculated that, even if the two species are sympatric in some areas, they are unlikely to hybridise.

Liu et al. (2021) used different iris colours to distinguish *Gonyosoma prasinum* and *G. coeruleum*. Poyarkov et al. (2025) observed individuals exhibiting either blue or yellow-green iris in *G. iadinum*, therefore, they suggested that iris colour should not be considered a reliable diagnostic characteristic within the *G. prasinum* species complex. However, based on the current limited observations, we found that the intraspecific variation in iris colour to be present only within *G. iadinum* (n = 10) and the iris colour is a stable characteristic within *G. prasinum* (n = 13) and *G. coeruleum* (n = 18). Therefore, we consider that iris colour cannot effectively distinguish all species of the *G. prasinum* species complex, but it is possible that iris colour could still be used as a diagnostic feature between *G. prasinum* and *G. coeruleum*.

Previously, *Boiga ochracea* was not formally recorded in China. However, some photography enthusiasts had captured species of *Boiga* with light brown colouration in Dehong Prefecture of Yunnan and they identified them as *B. ochracea* (now *B. multomaculata ochracea*). We checked some photos of so-called *B. ochracea* (*B. m. ochracea*) from Dehong Prefecture and found that they are likely to belong to *B. m. septentrionalis*. Therefore, we speculate that *B. m. ochracea* may not be distributed in Yunnan. As for whether *B. m. ochracea* is distributed in other parts of China, such as Xizang Autonomous Region, we are uncertain at present. Nevertheless, in this study, we confirm the distribution of *B. m. septentrionalis* in China, based on one specimen collected from Yunnan.

## Supplementary Material

XML Treatment for Gonyosoma prasinum

XML Treatment for Boiga multomaculata septentrionalis

## Figures and Tables

**Table 1.** Localities, voucher information and GenBank accession numbers for the genus *Gonyosoma* and outgroup taxa used in this study.

**Taxa**	**Locality**	**Voucher**	**Accession**
*Gonyosoma boulengeri*	Guangdong, China	HSR18178	MW495261
*Gonyosoma boulengeri*	Mengzi, Yunnan, China	CHS242	MK201360
*Gonyosoma boulengeri*	Lao Cai, Vietnam	HS12084	MW495262
*Gonyosoma coeruleum*	Mengla, Yunnan, China	KIZ2019025	MZ322870
*Gonyosoma coeruleum*	Mengla, Yunnan, China	KIZ2019026	MZ322869
*Gonyosoma coeruleum*	Mengla, Yunnan, China	KIZ2019027	MZ322868
*Gonyosoma coeruleum*	Mengla, Yunnan, China	KIZ2019028	MZ322867
*Gonyosoma coeruleum*	Zhenyuan, Yunnan, China	KIZ20200729	MZ322866
*Gonyosoma coeruleum*	Menglian, Yunnan, China	KIZ20200904	MZ322865
*Gonyosoma frenatum*	China	YPX11027	KF669250
*Gonyosoma frenatum*	Huangshan, Anhui, China	HS11038	KF669251
*Gonyosoma frenatum*	Huangshan, Anhui, China	CHS138	MK201289
*Gonyosoma frenatum*	Huangshan, Anhui, China	CHS139	MK201290
*Gonyosoma hainanense*	Hainan, China	CHS243	MK201361
*Gonyosoma hainanense*	Diaoluoshan, Hainan, China	HNU20190002	MW495259
*Gonyosoma hainanense*	Diaoluoshan, Hainan, China	ANU20190002	MW495258
*Gonyosoma hainanense*	Diaoluoshan, Hainan, China	ANU20190003	MW495260
*Gonyosoma iadinum*	Dak Lak, Vietnam	ZMMURe-18222	PV872147
*Gonyosoma iadinum*	Dak Nong, Vietnam	ZMMURe-18223	PV872146
*Gonyosoma iadinum*	Bao Lam, Lam Dong, Vietnam	ZMMURe-14426	PV872145
*Gonyosoma iadinum*	Khanh Hoa, Vietnam	ZMMURe-11551	PV872144
*Gonyosoma jansenii*	Sulawesi, Indonesia	—	DQ902113
*Gonyosoma margaritatum*	Sarawak, Borneo, Malaysia	—	KM870886
*Gonyosoma oxycephalum*	Dong Nai, Vietnam	ROM37622	KX694870
Gonyosoma cf. oxycephalum	Zamboanga, Mindanao, Philippines	KU321724	KC010343
*Gonyosoma prasinum*	Htamanthi, Sagaing, Myanmar	SEABRI2019120043	MZ322864
*Gonyosoma prasinum*	Htamanthi, Sagaing, Myanmar	SEABRI2019120075	MZ322863
*Gonyosoma prasinum*	Yingjiang, Yunnan, China	KIZ2025077	PX842325
*Coelognathus radiatus*	Wenshan, Yunnan, China	CHS556	MK201411
*Elaphe taeniura*	Recaokao, Sichuan, China	CHS203	MK201333

**Table 2.** Localities, voucher information and GenBank accession numbers for the genus *Boiga* and outgroup taxa used in this study.

**Taxa**	**Locality**	**Voucher**	**Accession**
*Boiga barnesii*	Sri Lanka	RAP0452	KC347469
*Boiga beddomei*	Mahabaleswar, Maharashtra, India	CESS444	MT733294
*Boiga bourreti*	Kon Plong, Kon Tum, Vietnam	ZISP32786	MN962356
*Boiga ceylonensis*	Sri Lanka	RS­Y	KC347467
*Boiga cyanea*	—	CHS553	MK201410
*Boiga cynodon*	Palawan Islands, Philippines	—	KC010340
*Boiga dendrophila*	—	—	AF471089
*Boiga dightoni*	Peermed, Kerala, India	ZSI­CZRC­V­7541	OP948298
*Boiga drapiezii*	—	LSUHC7295	KX660482
*Boiga flaviviridis*	Meghamalai, Tamil Nadu, India	—	MN508360
*Boiga forsteni*	Sri Lanka	RAP0540	KC347468
*Boiga irregularis*	—	—	FJ710794
*Boiga jaspidea*	Endau­-Rompin, Johor, West Malaysia	LSUHC7656	KX660484
*Boiga kraepelini*	Huangshan, Anhui, China	CHS115	MK201272
*Boiga multomaculata multomaculata*	Java, Indonesia	NK2708	ON932778
*Boiga multomaculata multomaculata*	Kanchanburi, Thailand	PT3083	ON932782
*Boiga multomaculata multomaculata*	Petchaburi, Thailand	PT3084	ON932783
*Boiga multomaculata ochracea*	Yin Ma Bin, Sagaing, Myanmar	CAS215390	MN962367
*Boiga multomaculata ochracea*	Ayeyarwady, Myanmar	SMF103792	ON932784
*Boiga multomaculata ochracea*	Magwe, Kayin, Myanmar	CAS243048	ON932776
*Boiga multomaculata ochracea*	Tha Baik Kyin, Mandalay, Myanmar	CAS216182	ON932770
*Boiga multomaculata septentrionalis*	Nagaland, India	WIIADR856	ON932788
*Boiga multomaculata septentrionalis*	Moenyin, Kachin, Myanmar	CAS241150	ON932772
*Boiga multomaculata septentrionalis*	Moenyin, Kachin, Myanmar	CAS241272	ON932773
*Boiga multomaculata septentrionalis*	Moenyin, Kachin, Myanmar	CAS241550	ON932774
*Boiga multomaculata septentrionalis*	Khandi, Sagaing, Myanmar	SMF106288	ON932771
*Boiga multomaculata septentrionalis*	Yingjiang, Yunnan, China	KIZ2025020	PX842326
*Boiga nigriceps*	—	LSUHC7020	KX660485
*Boiga nuchalis*	Coorg, Karnataka, India	CESS003	MT733270
*Boiga quincunciata*	Putao, Kachin, Myanmar	CAS221434	KX660451
Boiga cf. ranawanei	Sri Lanka	RAP0450	KC347466
*Boiga schultzei*	Estrella, Narra, Palawan, Philippines	KU327776	MN962368
*Boiga siamensis*	O’lakmeas, Pursat, Cambodia	LSUHC8502	KX660487
*Boiga stoliczkae*	Zhemgang, Bhutan	WIIADR858	OQ079990
*Boiga thackerayi*	Koyna, Maharashtra, India	BNHS2371	MN508359
*Boiga trigonata*	Sri Lanka	RS­143	KC347475
*Boiga westermanni*	India	—	MG428713
*Telescopus tripolitanus*	Mauritania	BEV9377	JX315531
*Toxicodryas pulverulenta*	—	CAS220642	KX660460

**Table 3.** Measurements (in mm) and scale counts of the specimens of *Gonyosoma prasinum* and *Boiga multomaculata septentrionalis* from China (for abbreviations, see Material and methods).

	***Gonyosoma prasinum*** **KIZ2025077** **Male**	***Boiga multomaculata septentrionalis*** **KIZ2025020** **Male**
SVL	558	870
TaL	159	166
TL	717	1036
TaL/TL	0.223	0.16
HL	24.0	20.2
HW	11.9	12.8
ED	3.9	3.7
Lor	1/1	1/1
PrO	1/1	1/1
PoO	2/2	2/2
SL	9/9	8/8
IL	10/10	11/11
SL-E	4–6/4–6	3–5/3–5
AT	2/1	2/2
PT	2/1	2/3
ASR	19	19
MSR	19	19
DSR	13	15
KAD	3	0
KMD	5	0
KPD	3	0
CLP	Undivided	Undivided
VEN	207	222
SC	99	96

## References

[B13634175] Che J, Jiang K, Yan F, Zhang Y (2020). Amphibians and reptiles in Tibet — Diversity and evolution.

[B13633318] David P, Campbell P, Deuti K, Hauser S, Luu V, Nguyen T, Orlov N, Pauwels O, Scheinberg L, Sethy P, Smits T, Teynié A, Vogel G (2022). On the distribution of *Gonyosoma
prasinum* (Blyth, 1854) and *Gonyosoma
coeruleum* Liu, Hou, Ye Htet Lwin, Wang & Rao, 2021, with a note on the status of *Gonyosoma
gramineum* Günther, 1864 (Squamata: Serpentes: Colubridae). Zootaxa.

[B13634231] Guo P, Che J (2024). Snakes in Qinghai-Xizang Plateau.

[B13634066] Köhler Gunther, Charunrochana Panupong, Mogk Linda, Than Ni, Kurniawan Nia, Kadafi Ahmad, Das Abhijit, Tillack Frank, O'Shea Mark (2023). A taxonomic revision of *Boiga
multomaculata* (Boie, 1827) and *B.
ochracea* (Theobald, 1868), with the description of a new subspecies (Squamata, Serpentes, Colubridae). Zootaxa.

[B13633240] Liu Shuo, Hou Mian, Lwin Ye Htet, Wang Qiaoyan, Rao Dingqi (2021). A new species of *Gonyosoma* Wagler, 1828 (Serpentes, Colubridae), previously confused with *G.
prasinum* (Blyth, 1854). Evolutionary Systematics.

[B13634091] Liu Shuo, Wang JiShan, Hou Mian, Zhang Liang, Wang Qiaoyan, Zong Chunmiao, Zhou Jiang, Rao Dingqi, David Patrick, Vogel Gernot (2025). A new species of the genus *Hebius* (Squamata, Natricidae), previously confused with *H.
boulengeri* (Gressitt, 1937). ZooKeys.

[B13633380] Poyarkov N, Bragin A, Idiiatullina S, Tran T, Le D, David P, Nguyen T (2025). A new species of the *Gonyosoma
prasinum* species complex (Squamata: Serpentes: Colubridae) from Indochina. Zootaxa.

[B13634080] Thompson Julie, Higgins Desmond, Gibson Toby (1994). CLUSTAL W: improving the sensitivity of progressive multiple sequence alignment through sequence weighting, position-specific gap penalties and weight matrix choice. Nucleic Acids Research.

[B13634043] Wallach V, Williams K, Boundy J (2014). Snakes of the world: A catalogue of living and extinct species.

[B13799131] Wallach Van, Midtgaard Rune, Hsiao Emma (2024). Revalidation of the Arboreal Asian Snake Genera *Gonyophis* Boulenger, 1891; *Rhynchophis* Mocquard, 1897; and *Rhadinophis* Vogt, 1922, with Description of a New Genus and Tribe (Squamata: Serpentes: Colubridae). Diversity.

[B13634106] Yang D, Rao D (2008). Amphibia and Reptilia of Yunnan.

